# Public social trust and acceptance of task shifting to hospital paramedics as a response to physician shortages

**DOI:** 10.1371/journal.pone.0355197

**Published:** 2026-08-27

**Authors:** Satoshi Kondo, Shuhei Ichikawa, Shuji Hashimoto, Masashi Izumiya, Masato Eto

**Affiliations:** 1 Department of Medical Education Studies, International Research Center for Medical Education, Graduate School of Medicine, The University of Tokyo, Tokyo, Japan; 2 Department of Medical Education, Graduate School of Medicine, University of Toyama, Toyama, Japan; 3 Center for Medical Education and Career Development, Graduate School of Medicine, University of Toyama, Toyama, Japan; 4 Department of General Medicine, Mie University Graduate School of Medicine, Mie, Japan; 5 Hashimoto General Clinic, Mie, Japan; 6 Clinical Simulation Center, The University of Tokyo Hospital, Tokyo, Japan; 7 Department of Gastroenterology, The University of Tokyo Hospital, Tokyo, Japan; Jordan University of Science and Technology, JORDAN

## Abstract

**Background:**

Physician shortage is a crucial global issue. Some countries have introduced task shifting to expand paramedics’ roles. This expansion requires citizens’ social trust in paramedics in general. During medical emergencies, citizens must allow treatment by paramedics, with whom they often have no prior relationship. Although social trust is vital in medical emergencies, its level among hospital paramedics is unclear. This study aimed to evaluate citizens’ social trust in hospital paramedics and their acceptance of the paramedics’ medical practices, as well as the impact of educational outreach regarding hospital paramedics.

**Methods:**

In this within-participant pre–post assessment, citizens completed a web-based questionnaire distributed online to assess social trust in hospital paramedics before and after receiving information about hospital paramedics. Social trust was assessed using a validated questionnaire, and acceptance of paramedic medical practices was evaluated using a five-point Likert scale. Descriptive analyses were performed to assess social trust and acceptance of paramedic medical practices. The impact of educational outreach was evaluated using mean differences and corresponding confidence intervals.

**Results:**

A total of 630 participants were included. Citizens’ social trust in hospital paramedics and their acceptance of paramedic medical practices were high. After educational outreach regarding hospital paramedics, acceptance of their practices showed a statistically significant but modest improvement, whereas social trust did not show a statistically significant change.

**Conclusions:**

Society may already accept hospital paramedics’ activities because social trust in ordinary paramedics has already been established. Task shifting to allied healthcare professionals to address physician shortages may require credible certification and education systems to gain high social trust and acceptance of hospital paramedics.

## Introduction

Recently, the increasing frequency and impact of disasters have received considerable global attention. The annual number of reported natural disasters increased several-fold, from approximately 100 events per year in the 1970s to about 400 events per year since 2000 [1]. Moreover, since 1970, the cumulative death toll from weather-, climate-, and water-related disasters has exceeded 2 million [2].

In this context, healthcare system capacity has become a critical concern, particularly in relation to physician shortages. In the US, projections estimate a deficit of tens of thousands of physicians over the next decade, potentially surpassing 100,000 in the worst-case scenario [3]. Such a shortage of doctors could represent a vulnerability in disasters. In fact, GAR 2025 highlights that complex and protracted disasters potentially generate cascading impacts that increase disaster-related mortality and morbidity through underlying structural vulnerabilities within healthcare systems [4].

To address these challenges, task shifting to paramedics has emerged as a key strategy for mitigating physician shortages. In particular, in Asia—a region recognized as disaster-prone due to frequent and compound disasters—research into the deployment of paramedics represents an important strategy for addressing structural vulnerabilities within healthcare systems, including chronic physician shortages [5]. For example, in primary care settings, paramedic-led assessment and treatment of emergency cases can reduce physicians’ workload [6]. Although the roles, regulations, and laws governing paramedics vary across countries, their functions and professions have been evolving [7]. However, public acceptance and social trust in the expanded roles of paramedics remain insufficiently understood.

In fact, the role and scope of paramedics have expanded over the past decade, and the permitted medical practices under the Japanese Emergency Life-saving Technicians Act have been amended to adapt to changing medical circumstances [8], thereby addressing Japan’s limited medical resources. Given that the availability of emergency services is low at night and on weekends [9], expanding paramedics’ activities in various settings has been proposed as a reasonable approach and a new policy measure to address the physician shortage [10]. The expansion of paramedics’ roles is considered crucial not only in Japan but also in the US. In response to workforce shortages, an act has been enacted to provide hospitals with grants for recruiting and training paramedics [11]. Thus, the roles of paramedics working in hospitals, namely, hospital paramedics, have expanded. For social accountability, citizens may be provided with information and educational outreach about the expanded medical practices of hospital paramedics in hospital emergency departments. Without sufficient educational outreach to inform the public about these expanded roles, public acceptance of expanded paramedic practices and social trust in hospital paramedics may remain limited.

Trust in professions and experts is necessary in modern society [12,13]. It is a key aspect of the patient–physician relationship [14,15] and is therefore vital for medical practice [16]. Trust in physicians and other healthcare professionals, including paramedics, is likewise essential. Given that trust is built on interpersonal relationships, it has inherent risks [17]. In a medical emergency, a person must rely on a paramedic even in the absence of interpersonal trust. Consequently, social trust in paramedics becomes more important than interpersonal trust. In health care, social trust refers to the feeling of trust in a professional cluster—not one particular person—responsible for managing safety [18]. Furthermore, given the limited time in medical emergencies, the impact of social trust in paramedics may be more vital than that in other medical professionals.

The study of social trust in medicine is a developing area of research [19]. Kondo et al. previously explored social trust in medical students and citizens’ acceptance of medical practices performed by these students [20] and inferred that these values could be improved through educational outreach to inform citizens about the medical students’ certification and curriculum [20]. Given that both medical students and paramedics practice medicine under physicians’ supervision, social trust in hospital paramedics and public acceptance of their medical practices may be related. However, trust in hospital paramedics and the effect of educational outreach remain insufficiently explored [19], and the impact of educational outreach on improving the acceptance of medical practices by hospital paramedics is still unclear. Informing the public about hospital paramedics’ education and certification may foster their professional activities.

In this study, the primary objective was to explore the level of the citizens’ social trust in hospital paramedics and their acceptance of the medical practices performed by these healthcare professionals. The secondary objective was to evaluate the effect of educational outreach regarding hospital paramedics on the citizens’ trust and acceptance.

This study contributes to the existing evidence on educational outreach, particularly regarding public awareness of hospital paramedics’ certification and education curriculum. It also offers new insights that may be useful for professional societies and governments in addressing their social accountability for new policies. Moreover, understanding public acceptance and trust in hospital paramedics will help promote evidence-based policy making to address physician shortages. The findings of this study could also provide helpful evidence for countries and regions implementing task shifting to healthcare professionals other than hospital paramedics.

## Materials and Methods

### Research design

A within-participant pre–post assessment was conducted using a web-based survey (S1 Text) to measure citizens’ social trust in hospital paramedics and their acceptance of paramedic-initiated medical practices (e.g., blood sampling), both before and after receiving educational outreach about paramedics’ roles and training (S2 Text).

### Setting and data collection

This study was conducted from March 24 to March 28 of 2022. We requested an internet research company to distribute our web-based questionnaire and collect data with no missing data. The study population consisted of adult residents in the target prefectures who were recruited through the panel maintained by the research company.

### Participants and recruitment

We also hired an internet research company to recruit participants representing the general Japanese population. The inclusion criteria were adults aged 20 years or older residing in the prefectures of Tokyo, Kanagawa, Saitama, and Chiba. Stratified random sampling by sex, age, and prefecture of residence was employed, thereby ensuring sample representativeness.

The internet research company distributed recruitment notices to the monitors registered for answering questionnaires. Individuals who agreed to participate after reading an explanation of the study were enrolled.

The sample size was determined based on commonly accepted recommendations for sample size in questionnaire-based studies, including those informed by item number and factor structure [21]. For a questionnaire with 18 items, at least 300 participants were considered sufficient under conservative assumptions; the actual sample size was 630.

### Ethics approval and consent to participate

The study protocol was approved by the Research Ethics Committee of the Faculty of Medicine of the University of Tokyo on September 15, 2021 (review no. 2021162NI), the Clinical Research Ethics Review Committee of Mie University Hospital on October 6, 2021 (review no. U2021-032), and the Ethics Review Committee for Clinical and Epidemiological Research, University of Toyama on June 5, 2023 (review no. R2023042). All study procedures were conducted according to the principles of the Declaration of Helsinki. The survey was conducted while the first author was affiliated with the University of Tokyo, and ethical approval was granted by the University of Tokyo’s ethics committee prior to data collection. After the author joined the University of Toyama during the data analysis phase, additional ethical approval was obtained from the University of Toyama to ensure compliance with institutional requirements for continuing the research. This approval was not retrospective but was sought before any further research activities were conducted at the new institution.

All participants received an informed consent document and provided electronic informed consent by checking a box on the web to indicate their agreement to participate in this study anonymously. Consent responses were recorded and securely stored without retaining any personal identifiers. The electronic informed consent via a web-based checkbox was approved by the ethics committee. This study included only adults aged 20 years and older.

### Questionnaire

The questionnaire was divided into five sections. The first section explained the study and obtained participants’ informed consent. The second section collected participants’ demographic information. The third section contained questions to evaluate participants’ social trust in hospital paramedics and their acceptance of paramedic-initiated medical practices (S1 Text). The items assessing social trust and acceptance were adapted from a measurement tool developed by Nakayachi et al. in studies on trust and risk-related behaviors, for which validity has been previously established [22,23]. Furthermore, these items have been used in different populations, and a model based on these factors has been examined using structural equation modeling in our previous study on citizens’ perspectives on medical students [20]. The fourth section presented information about hospital paramedics to the participants as educational outreach (S2 Text). The information included details on their training methods, certification, roles, and scope of practice, and was presented in a written format within the web-based survey. The content was standardized across participants and designed to provide neutral and factual information. The educational material was developed by a multidisciplinary team with expertise in medical education. Full text of the educational material is provided in S2 Text. The final section re-evaluated social trust in hospital paramedics and acceptance of paramedic medical practices after the participants reviewed the provided information. The questions about social trust in hospital paramedics and acceptance of paramedic medical practices were answered using a 5-point Likert scale (from 1 as “completely disagree” to 5 as “completely agree”).

### Statistical analyses

We first conducted descriptive analyses to assess the participants’ demographic characteristics, including means and standard deviations for continuous variables and proportions for categorical variables. Then, using descriptive statistics, we assessed their level of social trust in hospital paramedics and their acceptance of paramedic medical practices. Next, paired analyses were conducted for the before–after comparisons. The mean changes and confidence intervals were calculated by subtracting before from after to evaluate the impact of educational outreach regarding hospital paramedics on the citizens’ social trust and acceptance. To account for multiple comparisons, the significance level was set at 0.01 (99% confidence interval) [24]. Finally, subgroup analyses were conducted to compare citizens’ acceptance of such practices according to their previous experience with hospital paramedic–initiated medical practices.

All statistical analyses were performed using R (version 4.4.3) and RStudio (version 2024.12.1). Descriptive statistics were calculated using the tableone package. Other analyses, including pre–post comparisons and subgroup analyses, were conducted using base R functions.

## Results

This study included 630 participants, all of whom answered the questionnaire. Table 1 presents the participants’ demographic characteristics.

**Table 1:** Participants’ demographic characteristics.

Overall	*N* = 630
**Sex, *n* (%)**	
**Male**	316 (50.2)
**Female**	308 (48.9)
**Others**	6 (1.0)
**Age in years, mean (SD)**	45.5 (15.1)
**Range, years**	20–82
**Prefecture of residence, no. (%)**	
**Saitama**	127 (20.2)
**Chiba**	126 (20.0)
**Tokyo**	251 (39.8)
**Kanagawa**	126 (20.0)
**Q1 Are you a nurse or a physician? *n* (%)**	
**1 No**	609 (96.7)
**2 Nurse**	17 (2.7)
**3 Physician**	4 (0.6)
**If Q1 = 2 or 3 (nurse or physician), then answer Q2**	
**Q2 Have you ever taught hospital paramedics on medical practices, such as emergency treatment, when working as a medical staff member? *n* (%)**	
**Yes**	11 (52.4)
**No**	10 (47.6)
**Q3 As a patient or a family member of a patient, have you ever regularly visited a hospital or clinic or been hospitalized in the past? *n* (%)**	
**Yes**	397 (63.0)
**No**	233 (37.0)
**Q4 As a patient or a family member of a patient, when you visited a hospital or a clinic, did you ever receive medical practices, such as emergency treatment, by hospital paramedics? *n* (%)**	
**Yes**	66 (10.5)
**No**	564 (89.5)

Table 2 shows the participants’ level of social trust in hospital paramedics and their acceptance of paramedic medical practices. Social trust in hospital paramedics was high and almost same as that for supervising physicians. The total and specific acceptance scores for hospital paramedics’ medical practice were also high. Baseline scores were skewed toward higher values, with a large proportion of participants reporting scores near the upper limit of the scale. This pattern suggests a potential ceiling effect. The Cronbach’s α coefficient was 0.93 for the trust factor (Questions 1–3) and 0.96 for the acceptance items (Questions 16–24).

**Table 2:** Participants’ level of social trust in hospital paramedics and their acceptance of medical practices performed by these paramedics.

Score category (number of items)	Mean	SD
**Total score of trust (3)**	12.00	2.59
**Hospital paramedics can be trusted.**	4.02	0.92
**Hospital paramedics can be relied upon.**	4.05	0.92
**Hospital paramedics can be entrusted.**	3.92	0.92
**Total score of trust in supervising physicians (3)**	11.80	2.65
**Supervising physicians can be trusted.**	3.98	0.93
**Supervising physicians can be relied upon.**	3.93	0.92
**Supervising physicians can be entrusted.**	3.90	0.93
**Acceptance of blood glucose measurement (finger-stick glucose) (1)**	3.89	0.94
**Acceptance of blood pressure measurement (1)**	4.04	0.90
**Acceptance of drip infusion (1)**	3.92	0.94
**Acceptance of medical interview (1)**	3.86	0.97
**Acceptance of chest compression (1)**	4.07	0.91
**Acceptance of auscultation (1)**	3.91	0.96
**Acceptance of intubation (1)**	3.81	1.00
**Acceptance of using automated external defibrillation (1)**	4.09	0.93
**Acceptance of fracture fixation (1)**	3.95	0.94

Questions were answered using a 5-point Likert scale (from 1 as “completely disagree” to 5 as “completely agree”).

Table 3 shows the results of the mean differences and their confidence intervals to compare the participants’ levels of social trust in hospital paramedics and their acceptance of paramedic medical practices before and after receiving information about hospital paramedics. These data were used to evaluate the impact of educational outreach. Acceptance scores for paramedic medical practices showed a slight but statistically significant improvement after the participants were informed about hospital paramedics (mean value, 35.56 to 36.56; mean change = 1.00, [99% CI: 0.62, 1.39]). The level of social trust in hospital paramedics did not improve (mean value, 12.00 to 12.14; mean change = 0.14, [99% CI: −0.01, 0.3]).

**Table 3:** Mean differences and 99% confidence intervals (CI) before and after reading information about hospital paramedics.

Score category (number of items)	Before^†^ mean (SD)	After^‡^ mean (SD)	Mean difference	99% CI
**Total score of trust (3)**	12.00 (2.59)	12.14 (2.59)	0.14	−0.01, 0.3
**Total score of trust in supervising physicians (3)**	11.80 (2.65)	12.02 (2.70)	0.21	0.07, 0.36
**Total score of acceptance of paramedic medical practices (9)**	35.56 (7.41)	36.56 (7.59)	1.00	0.62, 1.39
**Acceptance of blood glucose measurement (finger-stick glucose test)**	3.89 (0.94)	4.07 (0.90)	0.18	0.11, 0.25
**Acceptance of blood pressure measurement**	4.04 (0.90)	4.13 (0.92)	0.09	0.02, 0.16
**Acceptance of drip infusion**	3.92 (0.94)	4.03 (0.94)	0.10	0.03, 0.17
**Acceptance of medical interview**	3.86 (0.97)	3.99 (0.95)	0.14	0.07, 0.21
**Acceptance of chest compression**	4.07 (0.91)	4.13 (0.93)	0.06	−0.003, 0.12
**Acceptance of auscultation**	3.91 (0.96)	4.03 (0.95)	0.12	0.05, 0.18
**Acceptance of intubation**	3.81 (1.00)	3.96 (0.98)	0.15	0.08, 0.23
**Acceptance of using automated external defibrillation**	4.09 (0.93)	4.14 (0.93)	0.05	−0.01, 0.12
**Acceptance of fracture fixation**	3.95 (0.94)	4.07 (0.94)	0.12	0.05, 0.18

† Before reading information about hospital paramedics.

‡ After reading information about hospital paramedics.

Table 4 shows the results of subanalysis on the impact of the participants’ previous experience of medical practices performed by hospital paramedics on their acceptance of such practices. Trust in hospital paramedics did not significantly differ between participants with and without previous experience of receiving medical interventions from hospital paramedics (mean value, 12.52 vs. 11.94; mean difference = 0.58 [99% CI: −0.34, 1.50]). Likewise, acceptance scores for paramedic medical practices did not significantly differ (mean value, 36.76 vs. 35.42; mean difference = 1.34 [99% CI: −1.09, 3.77]).

**Table 4:** Subanalysis comparing the scores of citizens with and without previous experience of paramedic medical practices to determine its impact on their acceptance of such practices.

Score category (number of items)	Group I (*n* = 66) mean (SD)	Group II (*n* = 564) mean (SD)	Mean difference	99% CI
**Total score of trust (3)**	12.52 (2.70)	11.94 (2.57)	0.58	−0.34, 1.50
**Total score of trust in supervising physicians (3)**	12.09 (2.63)	11.77 (2.65)	0.32	−0.58, 1.22
**Total score of acceptance of paramedic medical practices (9)**	36.76 (7.03)	35.42 (7.45)	1.34	−1.09, 3.77
**Acceptance of blood glucose measurement (finger-stick glucose test)**	3.89 (1.05)	3.89 (0.92)	0.002	−0.36, 0.36
**Acceptance of blood pressure measurement**	4.21 (0.77)	4.02 (0.92)	0.19	−0.08, 0.46
**Acceptance of drip infusion**	4.00 (0.94)	3.91 (0.94)	0.09	−0.24, 0.41
**Acceptance of medical interview**	4.09 (0.87)	3.83 (0.98)	0.26	−0.04, 0.57
**Acceptance of chest compression**	4.15 (0.86)	4.07 (0.91)	0.09	−0.21, 0.38
**Acceptance of auscultation**	4.11 (0.99)	3.89 (0.96)	0.22	−0.12, 0.56
**Acceptance of intubation**	3.95 (1.01)	3.79 (0.99)	0.16	−0.19, 0.51
**Acceptance of using automated external defibrillation**	4.24 (0.88)	4.07 (0.94)	0.17	−0.14, 0.47
**Acceptance of fracture fixation**	4.11 (0.91)	3.94 (0.95)	0.17	−0.14, 0.48

Group I: Participants with previous experience of medical practices performed by hospital paramedics.

Group II: Participants without previous experience of medical practices performed by hospital paramedics.

## Discussion

This study revealed two main findings. First, citizens’ level of social trust in hospital paramedics and their acceptance of paramedic medical practices were high. Second, educational outreach to inform citizens about hospital paramedics was associated with a statistically significant but modest improvement in acceptance of their practices. In contrast, no statistically significant change was observed in social trust.

Citizens’ levels of social trust in hospital paramedics and acceptance of medical practices performed by these healthcare professionals were both high (Table 2). The scores obtained were higher than those of social trust in medical students and acceptance of blood sampling performed by such students in our previous study [20]. Furthermore, the level of social trust in hospital paramedics appeared higher than that of social trust in professionals from other fields [23]. Notably, social trust in hospital paramedics was also higher than that reported for highly trusted public services, such as Japanese railway companies known for their reliability. The reason could be the high quality of emergency services in daily life and the activities of paramedics in earthquakes, such as the Great East Japan Earthquake in 2011. Surprisingly, social trust in hospital paramedics (12.00) was nearly equal to that in supervising physicians (11.80). Before the study, we assumed that physicians had gained greater social trust than hospital paramedics because of physicians’ higher level of expertise and longer training years. However, the present findings did not support this assumption. These findings suggest that task shifting from physicians to paramedics may be acceptable to the public and provide important insights into task shifting to allied medical professions. Nevertheless, given that paramedics and allied medical professions are at a disadvantage compared with physicians in terms of expertise and training duration, strengthening their competence to meet high levels of trust and acceptance of medical procedures is essential.

The expansion of paramedic roles is also evident in the UK and the US, which, like Japan, are high-income countries. In these countries, paramedics are taking on increasingly broader roles in primary and emergency care. Models such as extended care paramedics (ECP) in the United Kingdom and mobile integrated healthcare (MIH) in the United States illustrate the expansion of paramedic roles beyond traditional emergency response. For example, in the UK, the effectiveness of task-shifted patient care provided by paramedics has been demonstrated [25]. Meanwhile, in the US, the expansion of paramedic roles has been promoted as a strategy to address the shortage of healthcare professionals. Furthermore, a previous study has described the development of community paramedicine models in high-income settings such as Canada [26]. Differences in healthcare delivery systems and the expansion of paramedic roles across countries may be influenced by national economic conditions, including gross national income. In this context, the high levels of social trust and acceptance observed in this study may be understood within the framework of Japan’s well-developed emergency medical system.

Gaining information about hospital paramedics significantly but modestly improved the total score for citizens’ acceptance of paramedic medical practices (Table 3). Acceptance of blood glucose measurement (finger-stick glucose test), blood pressure measurement, drip infusion, medical interview, auscultation, intubation, and fracture fixation significantly increased after the participants received information about hospital paramedics. Acceptance of other medical procedures, such as chest compression and automated external defibrillation, also increased after participants received such information, but the increases were not statistically significant. Given that social trust and acceptance of medical procedures were already at extremely high levels, the ceiling effect could explain the failure to achieve a significant increase. This phenomenon may stem from public familiarity with procedures routinely performed by ordinary paramedics. In contrast with the previous study [20], the present study revealed that gaining information did not significantly improve social trust in hospital paramedics. This finding might be attributed to the ceiling effect resulting from the high level of social trust in hospital paramedics. This interpretation is supported by the distribution of baseline scores, which were skewed toward higher values. Although acceptance was consistently high across individual medical procedures, these analyses should be interpreted as exploratory in nature.

Moreover, citizens’ previous experience of medical practices performed by hospital paramedics did not appear to significantly impact their trust in hospital paramedics and acceptance of paramedic medical practices. In our subanalysis (Table 4), the trust and acceptance scores of citizens with previous experience of medical practices performed by hospital paramedics tended to be higher than the scores of those without such experience. Nonetheless, this result was not statistically significant. Interpersonal and social trust potentially reinforce each other through an amplification cycle [27], with interpersonal trust fostering patient autonomy [28]. Therefore, this higher level of acceptance possibly resulted from interpersonal trust in hospital paramedics built from previous interactions with hospital paramedics.

Given the high level of social trust identified in this study, social accountability may be important in policymaking and system design for hospital paramedics. While a high level of trust in hospital paramedics can be advantageous when performing medical practices, excessive trust may also pose risks for citizens, hospital paramedics, and supervising physicians [29]. Citizens who are overly trusting may pay less attention to checking whether the medical interventions provided by hospital paramedics are within their capabilities [13]. In Japan, the prehospital experience of hospital paramedics varies widely [30]. Therefore, mechanisms to support appropriate professional autonomy, such as the recognition and certification of hospital paramedics’ competencies by academic societies, professional organizations, and the government, may be considered. Through this, citizens may be able to make informed decisions based on these certifications. Furthermore, professional institutions, hospital paramedics, and supervising physicians could help inform citizens and society about hospital paramedics and their educational requirements. This may contribute to social accountability and has been emphasized in medical education. In medical education, social accountability has already led to the establishment of a common certification system for medical schools [31]. Therefore, implementing a common certification and education system for hospital paramedics may be beneficial in addressing the high level of trust in them.

Trust is the foundation of interprofessional collaboration [32,33]. Paramedics have pointed out the importance of trust from physicians [34]. When misunderstanding arises, interprofessional cooperation could be hindered [35]. Therefore, physicians’ trust in paramedics warrants investigation from this perspective.

This study has some limitations. First, data were collected from participants’ answers to a questionnaire concerning social trust in hospital paramedics and the acceptance of their medical practices. However, citizens’ decisions in an actual emergency may differ from the study findings. To minimize this issue, we attempted to ask the participants to assume whether they would trust (or distrust) and accept (or decline) hospital paramedics’ medical practices in an emergency department. In other words, this study was designed to evaluate citizens’ authentic trust and acceptance of paramedic medical practices without psychological pressure and time constraints. Given this approach, our findings provide a valuable contribution to recognizing trust in paramedics and accepting their medical practices. Second, interpreting our findings requires consideration of Japan’s cultural context. The roles and activities of hospital paramedics in Japan are beginning to expand and develop. Consequently, even after being provided with information about hospital paramedics, the participants might have answered the questionnaire according to their knowledge of traditional, ordinary paramedics. Furthermore, the measurement and social desirability provided by academic authority in Japan may have positively impacted the layperson participants’ trust in paramedics and acceptance of paramedic medical procedures. Despite these limitations, our study findings are of scientific value and provide suggestions for accelerating the acceptance of medical practices executed by hospital paramedics.

## Conclusions

This study uncovered high levels of public social trust in hospital paramedics and acceptance of paramedic medical practices. Furthermore, educational outreach aimed at informing citizens about the education and certification of hospital paramedics did not significantly enhance social trust and resulted in only a modest improvement in acceptance levels, likely due to an already high baseline. These findings strengthen the notion that expanding hospital paramedics’ role may be a socially accepted and trusted strategy for addressing physician shortages.

To the best of our knowledge, this study is the first to explore social trust in hospital paramedics using a quantitative approach.

## Supporting information

S1 Text Questionnaire evaluating social trust in hospital paramedics. (PDF)

S2 Text Information for study participants about hospital paramedics. (PDF)
