# Supplementary material for: Public social trust and acceptance of task shifting to hospital paramedics as a response to physician shortages
Source: PLoS One. 2026 Aug 27;21(8):e0355197. doi: 10.1371/journal.pone.0355197 (PMC13521363; doi:10.1371/journal.pone.0355197)
Supplement: Supplementary file 1 [file pone.0355197.s001.pdf]

S1 Text. Questionnaire evaluating social trust in hospital paramedics

Please answer the following questions assuming you were a patient at a hospital or clinic who received emergency medical practices from hospital paramedics.

Hospital paramedics:

1. Can be trusted.
2. Can be relied upon.
3. Can be entrusted.
4. Share the same point of view as mine.
5. Understand my feelings.
6. Priorities match mine.
7. Have high expertise.
8. Are efficient.
9. Have abundant expert knowledge.
10. Try their best.
11. Work very hard.
12. Are passionate.
13. Can help ensure patient safety.

14. Can eliminate danger to patients.

15. Contribute to the prevention of medical errors.

It is acceptable for hospital paramedics to perform:

16. Blood glucose measurements (finger stick glucose).

17. Blood pressure measurements.

18. Drip infusions.

19. Medical interviews.

20. Chest compressions.

21. Auscultation.

22. Intubation.

23. Automated external defibrillation.

24. Fracture fixation.

Supervising physicians

25. Can be trusted.

26. Can be relied upon.

27. Can be entrusted.

*Questions 1–3 measured citizens' trust in hospital paramedics, 4–6 measured perception of value similarity with hospital paramedics, 7–9 measured perception of ability, 10–12 measured perception of motivation, 13–15 measured perception of risk reduction, 16–24 measured acceptance of specific medical practices, and 25–27 measured trust in supervising physicians. All questions were answered using a 5-point Likert scale (1 = completely disagree and 5 = completely agree).
