# Supplementary material for: Public social trust and acceptance of task shifting to hospital paramedics as a response to physician shortages
Source: PLoS One. 2026 Aug 27;21(8):e0355197. doi: 10.1371/journal.pone.0355197 (PMC13521363; doi:10.1371/journal.pone.0355197)
Supplement: Supplementary file 2 [file pone.0355197.s002.pdf]

S2 Text. Information for study participants about hospital paramedics

Please answer the questionnaire about hospital paramedics after reading the following information concerning the certification examination and education necessary to become a hospital paramedic.

Do you think paramedics only perform medical practices and duties inside ambulances?

In fact, paramedics can work in hospitals and clinics and perform emergency medical practices on critically ill patients.

In the past, paramedics provided emergency medical practices before the patient arrived at a hospital or clinic. However, the Emergency Life-saving Technicians Act in Japan has been revised to allow paramedics to work in hospitals and clinics under the supervision of physicians. For example, hospital paramedics are engaged in coordination efforts between emergency medical institutions through a project to strengthen the system for receiving emergency patients in the Tokyo Metropolitan Government.

Certification of paramedics is required to perform medical practice in hospitals and clinics as hospital paramedics. First, they must graduate from a university or professional training college with a paramedic training course or work as a firefighter and complete the course at a paramedic training center. Then they must pass the National Paramedics Examination. This examination includes

questions about emergency medical care-related laws and emergency procedures for disease and injury performed by paramedics, such as patient observation, drip infusion, artificial ventilation, and chest compressions. Additionally, it also covers medical knowledge about the structure and function of the human body; disease causes, recovery, and prevention; and pharmaceuticals. After passing the National Paramedics Examination, ordinary paramedics can provide emergency medical practices to critically ill patients before arrival at hospitals or clinics.

Hospital paramedics perform their duties under the supervision of physicians. In particular, some emergency procedures, such as injecting drugs, infusion, and intubation for artificial ventilation, require specific directions from the supervising physicians. Moreover, hospital paramedics must receive training in medical safety administration and other areas before they can work in hospitals and clinics and perform emergency medical practices. Hospitals and clinics employing hospital paramedics usually organize committees of hospital paramedics to ensure the safe performance of medical practices.

Appendix II: Information for study participants about hospital paramedics (Japanese version)

以下の救急救命士の受ける試験と救急救命士のうける講習に関する説明をお読みいただき、アンケートにお答えください。

　みなさんは、救急救命士が救急車の中だけで活動すると思っていませんか？
　実は、救急救命士は病院やクリニックで勤務し、重症の患者さんに救急救命処置を行うことができます。以前は、重症の患者さんが病院やクリニックなどに運ばれるまでの間に、救急救命士が処置などを行ってきましたが、病院や診療所で医師の指示の下に救急救命士が活動できるように法律(救急救命士法)が改正されました。東京都救急搬送患者受入体制強化事業により、病院やクリニックで救急救命士が、救急医療機関の調整業務などに従事している例があります。
　救急救命士として病院やクリニックで働くためには、救急救命士の資格を得る必要があります。まず、救急救命士の国家試験を受験する資格を得るために、救急救命士の養成課程を持つ大学や専門学校などを卒業するか、消防官として勤務実績を積み、救急救命士養成所で講習を受けるなどの条件があります。次に、救急救命士の国家試験に合格しなくてはなりません。救急救命士国家試験では、救急医療に関する法律や救急救命士が行う傷病者の観察・点滴・人工呼吸や心臓マッサージなどの処置、救急に関わる病気や怪我だけでなく、人体の構造や機能、病気の原因・回復・予防、医薬品に関する知識など医学知識についても出題されます。救急救命士国家試験に合格することで、重症の患者さんが病院へ運ばれるまでの間に、救急救命士として救急救命処置を行うことができます。
　病院やクリニックで勤務する救急救命士は、医師の監督の下で活動します。特に、いくつかの救急救命処置（薬剤投与、点滴、人工呼吸のための気管内挿管など）は、医師の具体的な指示を受けることが必要です。さらに、病院やクリニックで勤務し救急救命処置を行うために、救急救命士は、医療の安全管理などに関する講習を受ける必要があります。また、救急救命士が勤務する病院やクリニックでは、救急救命士の活動が安全に行われるために救急救命士に関する委員会を設置する必要があります。
